# Platelet Dysfunction in Thrombosis Patients Treated with Vitamin K Antagonists and Recurrent Bleeding

**DOI:** 10.1371/journal.pone.0064112

**Published:** 2013-05-28

**Authors:** Paola E. J. van der Meijden, Annemieke C. Bouman, Marion A. H. Feijge, René van Oerle, Henri M. H. Spronk, Karly Hamulyák, Arina J. ten Cate-Hoek, Hugo ten Cate, Johan W. M. Heemskerk

**Affiliations:** 1 Department of Biochemistry, Cardiovascular Research Institute Maastricht (CARIM), Maastricht University Medical Center, Maastricht, The Netherlands; 2 Laboratory for Clinical Thrombosis and Haemostasis, Department of Internal Medicine, Cardiovascular Research Institute Maastricht (CARIM), Maastricht University Medical Center, Maastricht, The Netherlands; 3 Haemostasis Laboratory, Department of Internal Medicine, Maastricht University Medical Center, Maastricht, The Netherlands; Julius-Maximilians-Universität Würzburg, Germany

## Abstract

**Background:**

Recurrent bleeding can complicate the treatment of thrombosis patients with vitamin K antagonists (VKA), even at a well-regulated level of anticoagulation. In this proof-of-principle study, we investigated whether alterations in platelet function or von Willebrand factor (vWf) contribute to a bleeding phenotype in these patients.

**Methods:**

In this case-control study 33 well-regulated patients without bleeding events (controls) and 33 patients with recurrent bleeding (cases) were retrospectively included. Thrombin generation and vWf were determined in plasma. Platelet function was assessed by light transmission aggregometry and flow cytometry using a validated panel of agonists.

**Results:**

Thrombin generation was similarly reduced in controls and cases, in comparison to normal plasma. Plasma vWf level was above the normal range in 85% of controls and 67% of the cases. vWf activity was similarly increased in all patients in comparison to healthy volunteers. Platelet aggregation was in the normal range for almost all patients irrespective of the type of agonist. However, in response to a low collagen dose, platelets from 21% of controls and 27% of cases showed diminished responses. Agonist-induced secretion of alpha- and dense-granules or integrin αIIbβ3 activation were affected in platelets from neither controls nor cases.

**Conclusion:**

Recurrent bleeding in well-controlled patients on VKA therapy is not explained by anti-hemostatic changes in platelet or vWf function.

## Introduction

Anticoagulation therapy with vitamin K antagonists (VKA) is effective in the prevention and treatment of thrombotic complications, both in the venous and arterial vascular system. In the Netherlands, patient treatment with VKA is currently with either acenocoumarol (80%) or phenprocoumon (20%), both with a similar mechanism of action. To achieve a controlled level of anticoagulation, Dutch patients on VKA are monitored by regional the Thrombosis Services. This monitoring consists of regular (every 2–3 weeks) measurement of the international normalized ratio (INR) of the prothrombin time. Following guidelines of the Federation of Dutch Thrombosis Services, prior to the start of treatment, patients are assigned to INR target ranges of either 2.5–3.5 or 3.0–4.0 [1]. The nationwide aim of this guided and personalized therapy is to prevent not only recurrent thrombosis, but also bleeding complications due to over-anticoagulation [2].

Despite the permanent control of VKA therapy, acquired bleeding is still a major VKA treatment complication [3]. VKA treatment increases the risk of major bleeding events by 0.5% per year, with an absolute risk of 1–2% per year in the Netherlands [1]. In this country, major bleeding is defined by the Federation of Dutch Thrombosis Services as intracranial bleeding, joint bleeding or bleeding that leads to death, transfusion, surgery or hospitalisation [4]. Minor bleeding complications, comprising all other bleeding events, occur even more frequently with an estimated 15–20% per year [5]. Furthermore, there is a strong association between the intensity and duration of anticoagulation and the risk of bleeding. The bleeding incidence is highest during the first 90 days of treatment, and increases if INR values rise to >4.5 [6], [7]. In each patient, the quality of anticoagulation control, which is calculated as the time spent within the therapeutic INR range, is a key factor in predicting the risk of bleeding. Thus, patients appear to be best protected against bleeding, when their INR is >65% of the time within the therapeutic range. Nevertheless, also in these well-controlled patients, recurrent major bleeding is still observed [8]. Risk factors as far as known are age, gender and use of antithrombotic co-medication [6].

In individuals not on anticoagulants, the most common causes of bleeding disorders are abnormalities in level or function of von Willebrand factor (vWf) or platelets, both important components for the formation of a primary hemostatic plug at sites of vascular injury [9]. Typical for a primary hemostasis defect are excessive mucocutaneous bleeding events (i.e. easy bruising, prolonged and recurrent nosebleeds, or bleeding in the oral cavity), which can be more or less serious, depending on the defect [10], [11]. Registrations from the Thrombosis Services indicate that mucocutaneous bleeding is also a frequent treatment complication in well-controlled patients on VKA, suggesting that (partial) platelet or vWf dysfunction in these patients can explain the impaired hemostasis. This suggestion is supported by clinical studies demonstrating that combined treatment with VKA and antiplatelet drugs markedly increases the risk of bleeding complications [12], [13], [14]. A large cohort study of 11,480 patients with atrial fibrillation on VKA demonstrated a higher risk (hazard ratio of 1.47 within 90 days) of major bleeding in patients, when also prescribed dual antiplatelet therapy after myocardial infarction or percutaneous coronary intervention [15]. Together, partial platelet dysfunction may predispose for bleeding events, even in well-regulated patients treated with VKA, who are naïve for antiplatelet drugs.

In this paper, we hypothesize that alterations in platelet or vWf function contribute to the bleeding complications under a controlled VKA regimen. To investigate this, we performed a hypothesis-generating case-control study with well-regulated patients on VKA with either recurrent bleeding (cases) or no experienced bleeding (controls). Cases and controls were matched for age, gender and INR target range. Platelet function was analyzed in these patients using a panel of agonists, recommended as a diagnostic tool to identify congenital platelet function disorders in patients with bleeding symptoms. With a high positive (88%) and negative (84%) predictive value, this panel of agonists has proved its suitability for platelet function testing in a clinical setting [16].

## Methods

### Materials

Arachidonic acid was obtained from Chronolog (Havertown, PA, USA), epinephrine from Janssen Chimica (Geel, Belgium) and PAR1 peptide SFLLRN from (Bachem, Bubendorf, Switzerland). ADP and bovine serum albumin (BSA) came from Sigma (St. Louis, MO, USA), Horm collagen type I from Nycomed (Linz, Austria), ristocetin from American Biochemical and Pharmaceutical (Marlton, NJ, USA) and convulxin was purified from the venom of *Crotalus durissus terrificus* [17]. Fluorescein isothiocyanate (FITC)-labeled monoclonal antibody (mAb) against platelet-bound human fibrinogen was from WAK Chemie Medical (Steinbach, Germany), allophycocyanin (APC)-labeled mouse anti-CD63 mAb from Biolegend (San Diego, CA, USA), and FITC-labeled mouse anti-CD62P mAb from Beckman Coulter (Miami, FL, USA). Polyclonal rabbit anti-vWf (A0082) and horseradish peroxidase (HRP)-conjugated rabbit anti-vWf (P0226) antibodies came from Dako (Glostrup, Denmark).

### Ethics Statement

The study was approved by the Medical Ethics Committee of the Maastricht University Medical Center. All patients gave written informed consent for participation.

### Patient Selection

A hypothesis-generating case-control study was designed with 66 patients, who were on chronic treatment with VKA for at least 3 years, and were continuously monitored by the local Thrombosis Service. Patient data were collected from registries of the Thrombosis Service, including sex, age, indication for VKA treatment, prescribed VKA and other medication, monitored INR (every 2–3 weeks), and bleeding complications. From the records, at first patients were selected, who had a well-regulated level of anticoagulation according to the guidelines of the Federation of Dutch Thrombosis Services; an INR of either 2.5–3.5 or 3.0–4.0, depending on the indication for VKA therapy [18]. The selection implied that the INR was ≥65% of the time below the upper limit of the target range. The time in range was determined by calculating the fraction of INR measurements that were below the upper limit of the target range. The treatment period was confined from January 2007 to January 2010.

Among these patients, individuals were then retrospectively identified, who in spite of the controlled anticoagulation: (1) had experienced two or more minor or major bleeding events in this period, or (2) did not have any bleeding at all. Classification of bleeding events was according to the registration list of the Federation of Dutch Thrombosis Services [4]. Recorded bleeding events (minor and major) were: macroscopic haematuria, nosebleeds lasting >30 min and/or requiring treatment, menorrhagia, skin bleeds >10 cm in diameter, muscle/joints bleeds, bleedings of the digestive tract, conjunctival or intraocular bleeds, other bleedings in a critical area or organ and fatal bleeding. Patients with single bleeding events were not included, as this was considered to be accidental. Well-regulated patients identified with recurrent bleeding (cases) or without bleeding events (controls), were invited to participate in the case-control study.

### Patient Cases and Controls

The patient cases with recurrent bleeding events met one of the following criteria: (a) at least three months preceding every bleeding the INR was ≥65% of the time below the upper limit of the target range, or (b) at least three months preceding at least one of the bleedings the INR was ≥65% of the time below the upper limit of the target range ánd the last INR measured before the bleeding was below the upper limit of the target range. These criteria were applied to exclude bleeding complications as a consequence of incidental high INR values. The control patients had an INR, which was ≥65% of the time below the upper limit of the target range for 1 year, and were naïve for reported bleeding complications. Patient cases and controls were matched for age (±5 years), gender and INR target range. Patients with the following conditions were not included: age <18 years; use of antiplatelet drugs or other anticoagulant drugs than VKA; known platelet disorders; active malignancy; pregnancy; liver or kidney disease.

### Blood Collection and Preparation of Platelets and Plasma

Venous blood was drawn with a Vacutainer 21-gauge needle (Becton Dickinson, Plymouth, UK) and collected into 3.2% (w/v) citrate Vacuette tubes (Greiner Bio-One, Frickenhausen, Germany). Platelet-rich plasma (PRP) was obtained by centrifuging the blood at 240 g for 15 min, and platelet-free plasma (PFP) by centrifuging PRP twice at 2630 g for 10 min. Platelet count was determined with a thrombocounter (Coulter Electronics, Luton, UK) and normalized with autologous PFP to 250×10^9^ platelets/L. Functional assays were performed within 4 hours. PFP aliquots were directly frozen and stored at −80°C until analysis. All patients had normal platelet counts.

### Thrombin Generation

Thrombin generation in PFP was measured using the calibrated automated thrombogram (CAT) method, employing a fluorogenic thrombin substrate (Thrombinoscope, Maastricht, The Netherlands), under standard conditions as described [19]. Measurements were conducted with 80 µL plasma in 120 µL total volume. Added to the plasma was 20 pM tissue factor with 4 µM phospholipid vesicles (phosphatidyl serine/phosphatidyl ethanolamine/phosphatidyl choline, 20∶20∶60). After 10 min preheating at 37°C, 20 µL fluorogenic medium (2.5 mM fluorogenic substrate, 87 mM CaCl_2_) was added to start the thrombin generation. Calibrated, first-derivative thrombin generation curves were automatically analyzed for lag time, thrombin peak height, and endogenous thrombin potential (ETP; area under the thrombin generation curve). Normal ranges were established by triggering plasma from 139 healthy subjects with 5 pM tissue factor [20].

### Antigen Levels and Ristocetin Cofactor Activity of vWf

Antigen levels of vWf (vWf:Ag) were measured in plasma by a homemade ELISA using the anti-vWf antibodies A0082 (for coating) and P0226 (for detection), and vWf purified from human plasma as a standard. The normal range was determined as described [21]. To assess vWf function in plasma, ristocetin cofactor activity (vWf:RCoF) was determined using the BC von Willebrand Reagent (Siemens, Marburg, Germany). Platelet agglutination was assessed by a coagulation analyzer (BCS XP, Siemens). The normal range of the ristocetin cofactor activity was verified in plasmas from 22 healthy volunteers.

### Light Transmission Aggregometry

Platelet aggregation was measured in normalized PRP using a dual Chronolog aggregometer (Havertown, PA, USA). Samples were stimulated with different agonists at doses recommended in studies aiming to detect platelet function disorders [16]: 1 mM arachidonic acid, 10 µM epinephrine, 5–10 µM ADP, 15 µM PAR1 peptide SFLLRN (TRAP6), 1–4 µg/mL Horm collagen type I, or 1.5 mg/mL ristocetin. Traces were analyzed for slope and maximal light transmission changes. Normal ranges of aggregation per agonist were assessed as described elsewhere [22].

### Flow Cytometry

Samples of PRP were diluted 1∶10 in Hepes buffer (10 mM Hepes, 136 mM NaCl, 2.7 mM KCl, 2 mM MgCl_2_, 0.1% glucose and 0.1% BSA; pH 7.45), and then activated: 10 µM ADP, 15 µM SFLLRN or 50 ng/mL convulxin. After 20 min, activated platelets were assessed for surface activation markers with FITC-labeled anti-CD62P mAb (1∶200), APC-labeled anti-CD63 mAb (1∶5), or FITC-labeled mAb against platelet-bound human fibrinogen, detecting activated integrin αIIbβ3 (1∶1) using a three-channel Accuri C6 flow cytometer (BD Biosciences, San Jose, CA, USA).

### Statistical Analysis

Data are given as median with interquartile ranges or as mean ± SD. Statistical analysis was performed using the Mann-Whitney *U* test or the independent samples *t* test, as appropriate. P-values <0.05 were considered statistically significant. GraphPad Prism 5.0 software was used for statistical and graphical purposes (GraphPad Software, San Diego, CA, USA).

## Results

### Patient Characteristics

In total 66 patients who were treated with VKA and monitored by the Thrombosis Service were included; 33 had experienced recurrent bleeding (cases) and 33 were without reported bleeding during three years (Table 1). The main indication for VKA therapy was atrial fibrillation, i.e. in 52% of controls and 64% of the cases. Other indications were heart disease (arrhythmia, heart valvular disease, cardiomyopathy, CABG), venous (deep vein thrombosis, pulmonary embolism) or arterial thrombosis (peripheral arterial disease). Diabetes mellitus was present in 3 controls and 4 cases. The majority of bleeding complications in the cases could be classified as minor, except for 1 urinary tract, 1 muscle and 2 major nose bleedings. Most frequently recorded was minor nose bleeding (n = 26), followed by minor conjunctiva bleeding (n = 22). The control and case groups were matched for mean age (73–77 years) and sex (64–61% male), as indicated in Table 1. Most of the patients were treated with acenocoumarol (controls 85%, cases 79%). Controls and cases were also matched for INR target ranges (76% at low intensity anticoagulation with INR 2.5–3.5).

**Table 1:** Characterization and bleeding events of the studied patients.

		Controls	Cases
***Inclusion***			
No bleeding		33	0
Bleeding	criteria 1	0	28 (84.8)
	criteria 2	0	5 (15.2)
***Bleeding localisation***			
		***minor/major***	***minor/major***
Urinary tract		0/0	10/1 (14.9)
Gastrointestinal tract		0/0	9/0 (12.2)
Nose		0/0	24/2 (35.1)
Skin		0/0	9/0 (12.2)
Muscle		0/0	0/1 (1.4)
Conjunctiva		0/0	22/0 (29.7)
***Characteristics***			
Age, mean (SD)		73.1 (9.4)	76.5 (7.3)
Male		21 (63.6)	20 (60.6)
INR target range	2.5–3.5	25 (75.8)	25 (75.8)
	3.0–4.0	8 (24.2)	8 (24.2)

Included were 66 patients on prolonged VKA medication, reported to be well-regulated by the Thrombosis Service. Controls comprised 33 patients without registered bleeding and the cases were 33 patients who experienced two or more bleeding complications. Shown are the numbers of specified bleeding complications and the patients characteristics used for matching. N (%).

### Similarly Suppressed Thrombin Generation Profiles in Plasmas from Controls and Cases

**Figure 1:**
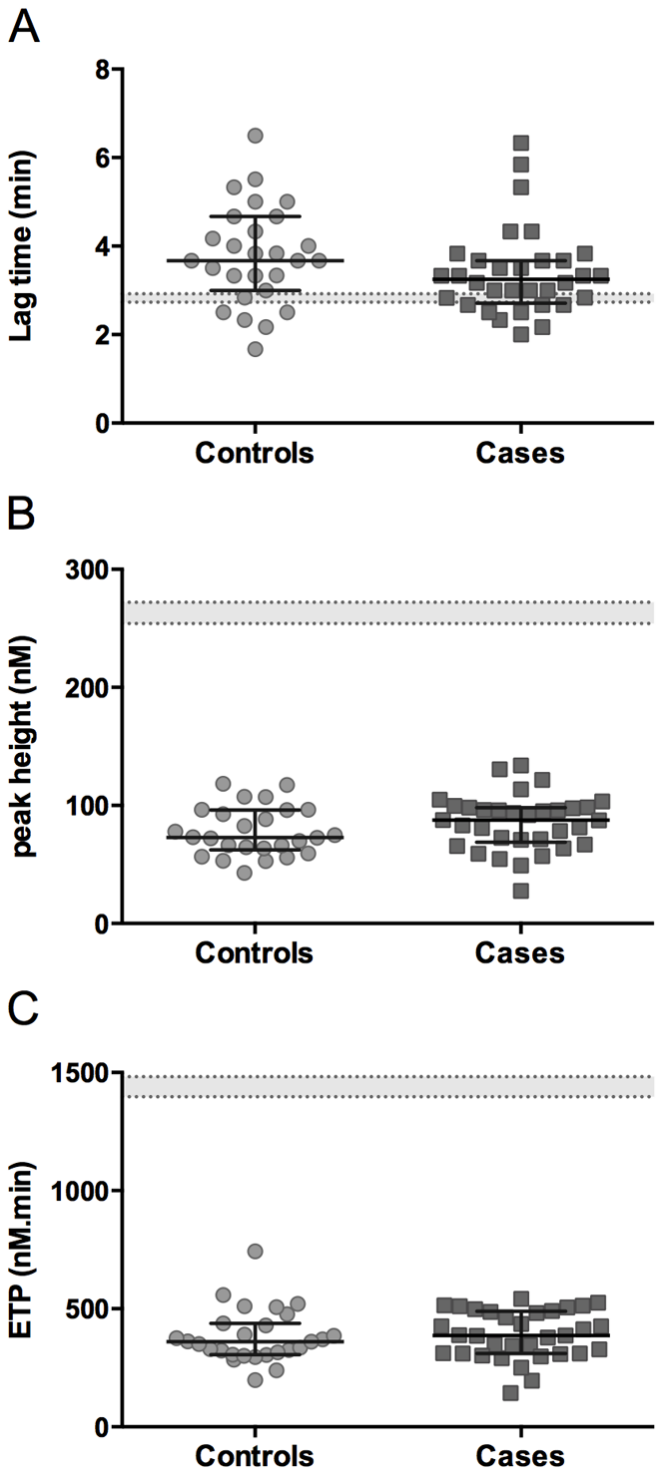
Thrombin generating capacity in plasma of the controls and cases. Thrombin generation was measured in plasma triggered with 20 pM TF using the CAT method. Curve parameters are lag time (A) and peak height (B) of thrombin generation, and endogenous thrombin potential (ETP) (C). Normal ranges (plasmas from healthy subjects) are represented by dotted lines. Indicated are medians with interquartile ranges.

At the day of blood collection, INR values of all patients were within the target range with a median INR of 2.9 (CI 2.7–3.0) and 3.0 (CI 2.7–3.1) for controls and cases, respectively. The efficacy of VKA treatment in both groups was also assessed by measurement of thrombin generation in plasma, as a capacitive test for the anticoagulant state. This was considered to be relevant, since conventional coagulation tests, like the prothrombin time and thus INR, monitor fibrin clotting when only <5% of the total amount of thrombin is generated. Plasma was triggered with a high concentration of 20 pM tissue factor to be able to measure thrombin generation under anticoagulation therapy. The lag time and the peak height of the thrombin generation curves were not significantly different between cases and controls (Figure 1A, B). The median thrombin-generating capacity (ETP) was similar for the controls and cases, i.e. 361 (CI 315–429) and 387 (CI 328–463) nM thrombin•min, respectively (Figure 1C). Thrombin peak heights and ETP levels of all patients (cases and controls) were reduced, when compared to the normal range of values with plasmas from untreated, healthy individuals (5 pM tissue factor). Together, this confirmed a similar degree of anticoagulation due to VKA therapy in the controls and cases.

### Increased Level and Activity of vWf in Plasmas from Controls and Cases

**Figure 2:**
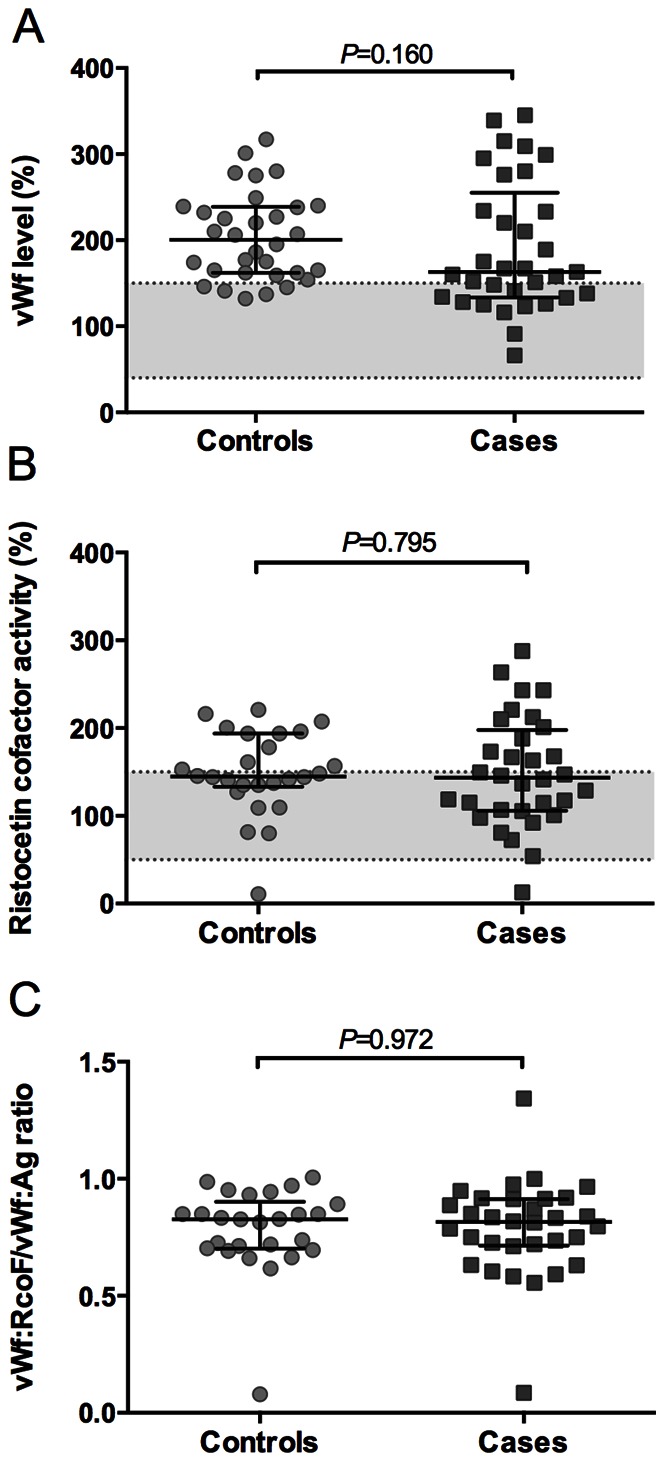
vWf levels and activity in controls and cases. Antigen levels (vWf:Ag, A) and ristocetin cofactor activity (vWf:RCoF, B) of vWf were determined in patients plasmas, and the ratio (C) was calculated. Normal ranges (plasmas from healthy subjects) are indicated by dotted lines. Data are shown as median levels with interquartile ranges.

Given that quantitative or qualitative abnormalities in vWf are among the most common causes of mild bleeding disorders [9], we assessed the plasma levels and activity of vWf in both groups. Strikingly, in comparison to normal plasma, vWf antigen levels were increased in 85% of controls and in 67% of the cases (Figure 2A). The increase was similar in cases and controls (*P* = 0.16). Furthermore, in 42% of the controls and 41% of the cases, vWf ristocetin cofactor activity was above the normal range (Figure 2B). Compared to healthy volunteers (median: 94%, CI 75–118%), vWf activity was significantly higher in both controls (median: 145%, CI 135–178%) and cases (median: 144%, CI 115–173%). By implication, vWf levels correlated well with the vWf ristocetin cofactor activity (r = 0.830 for controls and r = 0.883 for cases, *P*<0.001), while the vWf activity/antigen ratio was similar for both groups (Figure 2C).

### Platelet Activation Tendency in Controls and Cases

**Figure 3:**
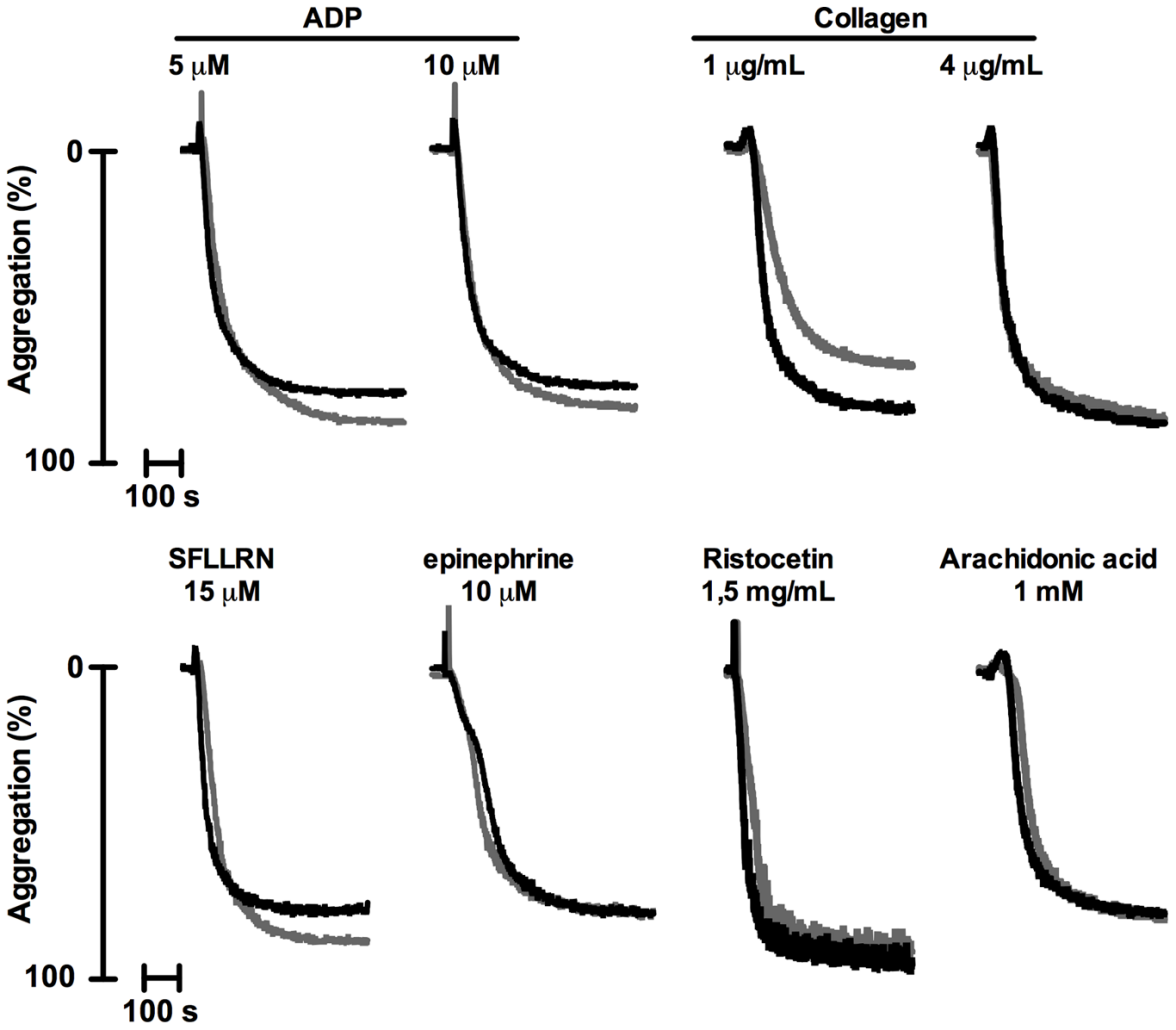
Platelet aggregation for controls and cases in response to a standard agonist panel. Aggregation in PRP (250×10^9^ plts/L) was determined in response to a streamlined panel of agonists: ADP (5 or 10 µM), collagen (1 or 4 µg/mL), SFLLRN (15 µM), epinephrine (10 µM), ristocetin (1.5 mg/mL) or arachidonic acid (1 mM). Representative aggregation traces are shown for controls (grey) and cases (black).

**Figure 4:**
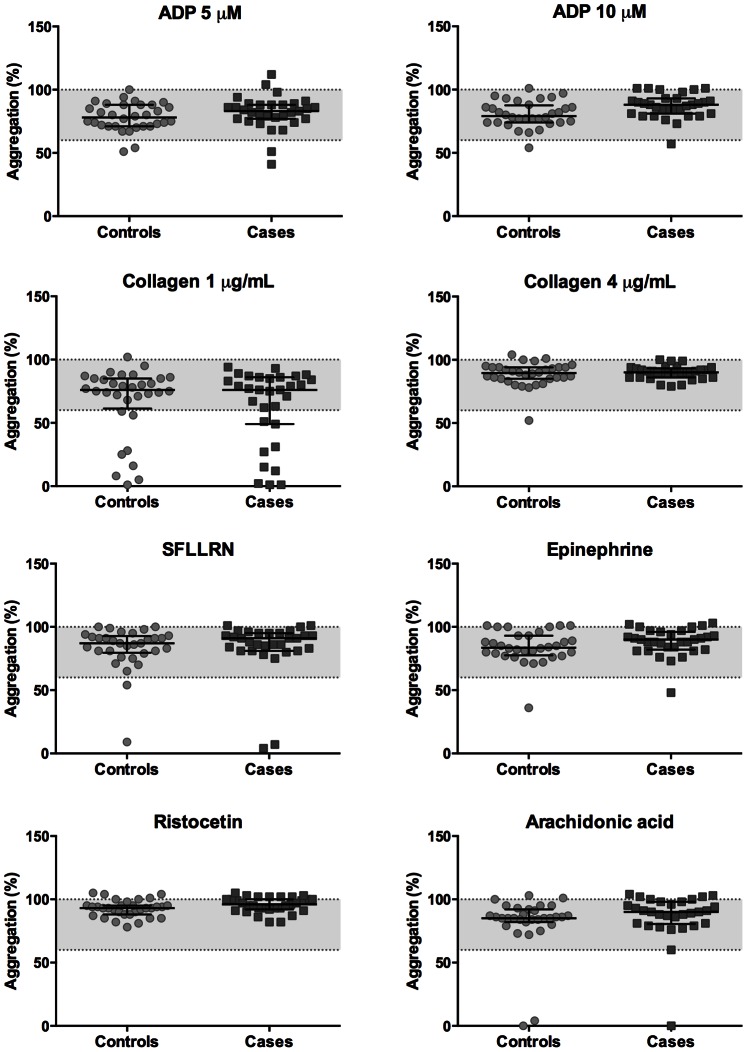
Maximal aggregation extent for all controls and cases. Quantification of the results depicted in figure 2. Maximal aggregation is shown (median with interquartile range). Normal ranges for platelets from healthy subjects are indicated by the dotted lines.

Platelet function was assessed in freshly isolated PRP from all patients by light transmission aggregometry (LTA), which is the golden standard for detecting abnormalities associated with a bleeding disorder [23]. A previously validated panel of six platelet agonists was used [16], to be able to detect the most frequent types of platelet function defects, i.e. aberrant ADP-induced Gi signaling, impaired thromboxane A_2_ production or signaling, or granule secretion defects [24]. These agonists were used, as recommended, at relatively high doses [16]: ADP (10 µM), epinephrine (10 µM), arachidonic acid (1 mM), PAR1 agonist SFLLRN (15 µM), collagen (4 µg/mL) or ristocetin (1.5 mg/mL). Normal aggregation patterns were obtained with PRP from the controls and cases (Figure 3). Quantification of the results indicated that the platelets from almost all patients showed normal aggregation responses in response to each agonist (Figure 4 and Tables S1 and S2). However, platelets from one of the controls and two cases did not respond to the PAR1 peptide. One of these cases also showed a slightly diminished epinephrine-induced aggregation. Platelets from two controls and one of the cases did not respond to arachidonic acid, which could later be attributed to aspirin intake. Ristocetin-induced agglutination was normal for PRP from all patient controls and cases, pointing to normal vWf interaction with platelets. Platelet responses following ADP (10 µM) or ristocetin stimulation were even higher in cases than in controls (Tables S1 and S2).

To substantiate these findings, aggregation of platelets was also measured in response to a lower dose of 5 µM ADP or 1 µg/mL collagen. No marked effects of the lower ADP dose were observed, but platelet activation with low collagen resulted in a reduced aggregation response in 7 controls and 9 cases (Figure 4). The difference between controls and cases was not statistically significant (*P* = 0.766).

**Figure 5:**
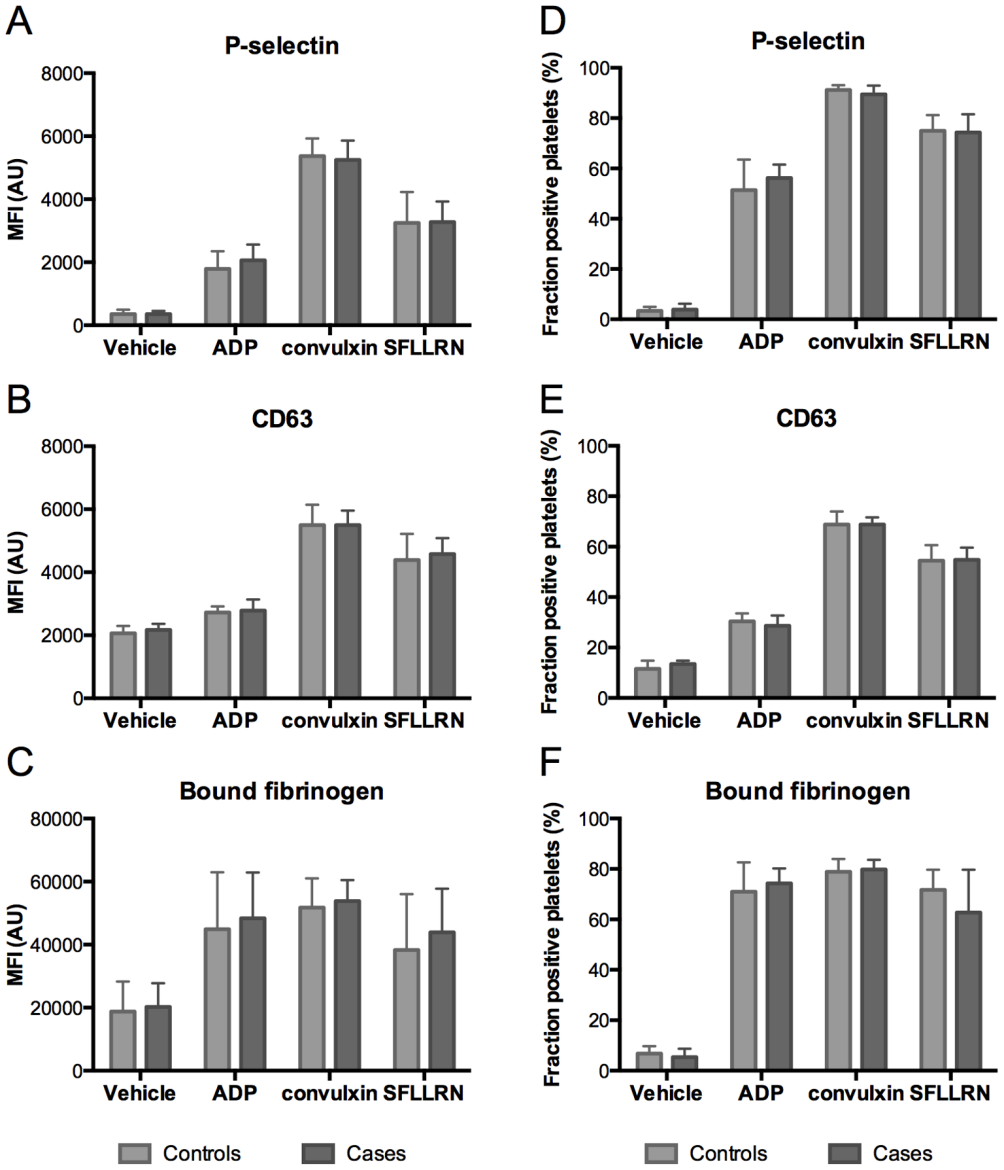
Agonist-induced secretion and integrin activation of platelets from controls and cases. PRP diluted in Hepes buffer was activated with 10 µM ADP, 50 ng/mL convulxin or 15 µM SFLLRN. Flow-cytometric detection of α-granule secretion using FITC-labeled P-selectin (A, D), dense-granule secretion with APC-labeled anti-CD63 (B, E), and platelet fibrinogen binding with FITC-labeled anti-fibrinogen mAb (C, F). Data are mean fluorescence intensity (MFI, left panels) and fractions of positive platelets (right panels). Medians with interquartile ranges.

In parallel, we measured the expression of platelet surface activation markers in response to a similar panel of agonists by flow cytometry: 10 µM ADP, 15 µM SFLLRN or 50 ng/mL convulxin (a non-fibrillar glycoprotein VI agonist, replacing collagen). In order to discriminate between different types of platelet secretion defects [25], secretion of α- and δ-granules was separately determined by the expression of P-selectin (CD62P) and CD63, respectively, with labeled antibodies. Secretion from either type of granules was most pronounced after platelet activation with convulxin, followed by PAR1 agonist SFLLRN and ADP (Figure 5A, B). No difference was measured between the platelets from patient controls and cases, regardless of whether the results were expressed as mean labeling fluorescence units or as percentages of positive platelets (Figure 5D, E). For full data, see Tables S3 and S4.

In line with the results obtained by LTA, binding of fibrinogen, reflecting integrin αIIbβ3 activation, was achieved with ADP, convulxin or SFLLRN. Also this parameter was not significantly different between controls and cases (Figure 5C, F). Taken together, although the platelets from incidental cases showed a low response to a particular agonist, statistically there was no difference in any of the platelet activation parameters between the patient cases and controls.

## Discussion

In individuals naïve for anticoagulant treatment, quantitative or qualitative abnormalities in platelets or vWf function are the most common causes of bleeding disorders. However, in this hypothesis-generating study, no differences in platelet function or vWf level or function could be found between patients with recurrent bleeding (cases) or without bleeding (controls), whose anticoagulant medication with VKA was well-controlled during a prolonged period. Thus, using a previously validated panel of agonists for the detection of platelet function defects, no significant differences were found in platelet aggregation tendency, platelet secretion or integrin activation between the two patient groups. Stated otherwise, in spite of the anticoagulant treatment (as confirmed by a reduced thrombin generation), the platelets of the majority of patients responded normally to most or all agonists.

A remarkable finding was that several controls and cases showed reduced platelet aggregation in response to the low dose of collagen. A major predictor of low collagen-induced platelet function is the T13254C polymorphism (rs1613662) in *GP6*, the gene encoding GPVI [26]. We found that the minor allele frequency of the *GP6*T13254C variant was 16.7% in the controls and 15.2% in the cases, and that this could not explain differences between the groups (van der Meijden, unpublished data, 2013).

Another new finding is that plasma levels of vWf and well as vWf ristocetin cofactor activity were above the normal range in the majority of case and control patients. This also makes it quite unlikely that the recurrent bleeding phenotype in the patient cases is related to aberrant vWf activity. The main indication for VKA therapy in both patient groups was atrial fibrillation; a condition that develops after longstanding increased arterial pressure and may associate with secondary endothelial damage and a thrombotic tendency. In line with the present results, other studies have shown that levels of vWf are raised in patients with atrial fibrillation, and that these are predictive for stroke and other vascular events [27], [28], [29].

On the other hand, recent association studies suggest that high vWf levels during anticoagulant treatment are not only predictive for thromboembolic events, but may also serve as a risk marker for major bleeding [30], [31]. While this is contra-intuitive with our knowledge of the roles of vWf in hemostasis, it may point to significant endothelial dysfunction and vessel wall abnormalities in part of the treated patients, and stipulate that the risk of bleeding is of a vascular rather than a blood-borne cause. In this respect, we repeat that in our study plasma levels of vWf for the cases and controls were similarly high.

In the present study, most patients were at advanced age; with a mean of 73 years for controls and 77 years for the cases. Age has been regarded as a risk factor, as higher bleeding rates have been reported in elderly people [7], [32], [33]. This has been ascribed to factors that are more common in elderly, such as co-morbidities, use of multiple drugs, reduced metabolic clearance of VKA and pathological changes in the blood vessels [34]. However, not all studies are in agreement with patient age as a risk factor [35], [36]. In a recent study with very old patients (≥80 years) on VKA, the frequency of bleeding complications was low with adequate management, indicating that age per se is not the main relevant risk factor for bleeding [37]. In our study, bleeders and non-bleeders were matched for these common risk factors.

Some weaknesses of our study have to be discussed. First, the majority of the patients (controls and cases) were treated with acenocoumarol, which has a shorter half-life (8–11 hours) and is therefore associated with less stable anticoagulation in comparison to phenprocoumon (half life 5–6 days). This means that short-term increases in INR, not detected by the Thrombosis Services, cannot be excluded for the patients on acenocoumarol. It is difficult to control for this factor though, since especially minor bleeding complications are often reported at a later point in time, as a result of which INR values at the time of bleeding are not always available. Secondly, samples tested are not drawn at the time of bleeding. Therefore, we cannot be completely certain that the composition of the blood is representative. Third, about one-fourth of the cases and controls were maintained at a INR target range of 3.0–4.0, *i.e.* slightly higher than the common target range of 2.5–3.5. The range 2.5–4.0 however does not translate to an increased bleeding risk and bleeding was not significantly more seen in patients within the higher range [8]. Fourth, in spite of recent improvements, bleeding assessment tools are still considered to be imperfect [38]. However, despite these limitations and the limited power of our study, the high similarity in platelet responses between cases and controls makes it unlikely that platelet dysfunction is a main cause of recurrent bleeding in well-controlled patients on VKA.

## Supporting Information

Table S1 **Maximal platelet aggregation for control and case patients with a standard agonist panel.** Maximal aggregation in PRP (250×10^9^ plts/L) was determined in response to a streamlined panel of agonists: ADP (5 or 10 µM), collagen (1 or 4 µg/mL), SFLLRN (15 µM), epinephrine (10 µM), ristocetin (1.5 mg/mL) or arachidonic acid (1 mM). Medians with interquartile ranges.
(DOC)

Table S2 **Rate of platelet aggregation for control and case patients in response to a standard agonist panel.** Slope of aggregation curves in PRP (250×10^9^ plts/L) was determined in response to a streamlined panel of agonists: ADP (5 or 10 µM), collagen (1 or 4 µg/mL), SFLLRN (15 µM), epinephrine (10 µM), ristocetin (1.5 mg/mL) or arachidonic acid (1 mM). Medians with interquartile ranges.
(DOC)

Table S3 **Agonist-induced secretion and integrin activation for platelets from controls and cases.** PRP diluted in Hepes buffer was activated with 10 µM ADP, 50 ng/mL convulxin or 15 µM SFLLRN. Flow-cytometric detection of α-granule secretion using FITC-labeled P-selectin, dense-granule secretion with APC-labeled anti-CD63 and platelet fibrinogen binding with FITC-labeled anti-fibrinogen mAb. Data represented as mean fluorescence intensity (MFI). Medians with interquartile ranges.
(DOC)

Table S4 **Agonist-induced secretion and integrin activation of platelets from controls and cases.** PRP diluted in Hepes buffer was activated with 10 µM ADP, 50 ng/mL convulxin or 15 µM SFLLRN. Flow-cytometric detection of α-granule secretion using FITC-labeled P-selectin, dense-granule secretion with APC-labeled anti-CD63 and platelet fibrinogen binding with FITC-labeled anti-fibrinogen mAb. Data represented as fractions of positive platelets. Medians with interquartile ranges.
(DOC)
