# Supplementary material for: Platelet Dysfunction in Thrombosis Patients Treated with Vitamin K Antagonists and Recurrent Bleeding
Source: PLoS One. 2013 May 28;8(5):e64112. doi: 10.1371/journal.pone.0064112 (PMC3665853; doi:10.1371/journal.pone.0064112)
Supplement: Table S1 — Maximal platelet aggregation for control and case patients with a standard agonist panel. Maximal aggregation in PRP (250×109 plts/L) was determined in response to a streamlined panel of agonists: ADP (5 or 10 µM), collagen (1 or 4 µg/mL), SFLLRN (15 µM), epinephrine (10 µM), ristocetin (1.5 mg/mL) or arachidonic acid (1 mM). Medians with interquartile ranges. (DOC) [file pone.0064112.s001.doc]

| **Agonist** | **Controls**  *max. aggregation (%)* | **Cases**  *max. aggregation (%)* | ***P*-values** |
| --- | --- | --- | --- |
| ADP 5 μM | 78.0 (71.0-88.0) | 83.0 (77.0-88.0) | 0.178 |
| ADP 10 μM | 79.0 (74.0-87.5) | 88.0 (81.0-93.0) | 0.009 |
| Collagen 1 μg/mL | 76.0 (61.3-85.0) | 76.0 (49.0-86.0) | 0.766 |
| Collagen 4 μg/mL | 89.5 (85.0-94.0) | 90.0 (86.0-93.3) | 0.723 |
| SFLLRN | 87.0 (79.5-92.8) | 91.0 (81.0-95.0) | 0.346 |
| Epinephrine | 83.5 (77.5-93.0) | 90.0 (82.0-96.0) | 0.102 |
| Ristocetin | 93.0 (88.0-95.0) | 96.0 (92.0-100) | 0.063 |
| Arachidonic acid | 85.0 (82.0-92.0) | 90.0 (80.5-98.0) | 0.122 |
