# Supplementary material for: Platelet Dysfunction in Thrombosis Patients Treated with Vitamin K Antagonists and Recurrent Bleeding
Source: PLoS One. 2013 May 28;8(5):e64112. doi: 10.1371/journal.pone.0064112 (PMC3665853; doi:10.1371/journal.pone.0064112)
Supplement: Table S2 — Rate of platelet aggregation for control and case patients in response to a standard agonist panel. Slope of aggregation curves in PRP (250×109 plts/L) was determined in response to a streamlined panel of agonists: ADP (5 or 10 µM), collagen (1 or 4 µg/mL), SFLLRN (15 µM), epinephrine (10 µM), ristocetin (1.5 mg/mL) or arachidonic acid (1 mM). Medians with interquartile ranges. (DOC) [file pone.0064112.s002.doc]

| **Agonist** | **Controls**  *aggregation slope (%/min)* | **Cases**  *aggregation slope (%/min)* | ***P*-values** |
| --- | --- | --- | --- |
| ADP 5 μM | 101 (86.0-117) | 110 (96.0-121) | 0.182 |
| ADP 10 μM | 107 (94.0-119) | 110 (99.0-129) | 0.194 |
| Collagen 1 μg/mL | 80.0 (48.8-95.5) | 72.0 (46.0-90.0) | 0.410 |
| Collagen 4 μg/mL | 110 (101-121) | 108 (103-124) | 0.947 |
| SFLLRN | 115 (95.3-136) | 127 (105-145) | 0.541 |
| Epinephrine | 68.0 (50.8-92.0) | 78.0 (48.0-88.0) | 0.862 |
| Ristocetin | 134 (120-147) | 142 (136-151) | 0.043 |
| Arachidonic acid | 90.0 (77.0-105) | 87.0 (74.8-105) | 0.680 |
