# Supplementary material for: Platelet Dysfunction in Thrombosis Patients Treated with Vitamin K Antagonists and Recurrent Bleeding
Source: PLoS One. 2013 May 28;8(5):e64112. doi: 10.1371/journal.pone.0064112 (PMC3665853; doi:10.1371/journal.pone.0064112)
Supplement: Table S3 — Agonist-induced secretion and integrin activation for platelets from controls and cases. PRP diluted in Hepes buffer was activated with 10 µM ADP, 50 ng/mL convulxin or 15 µM SFLLRN. Flow-cytometric detection of α-granule secretion using FITC-labeled P-selectin, dense-granule secretion with APC-labeled anti-CD63 and platelet fibrinogen binding with FITC-labeled anti-fibrinogen mAb. Data represented as mean fluorescence intensity (MFI). Medians with interquartile ranges. (DOC) [file pone.0064112.s003.doc]

| **Marker** | **Agonist** | **Controls**  *MFI (AU)* | **Cases**  *MFI (AU)* | ***P*-value** |
| --- | --- | --- | --- | --- |
| P-selectin | Vehicle | 356  (291-499) | 357  (298-456) | 0.915 |
|  | ADP | 1,793  (1,407-2,351) | 2,064  (1,549-2,561) | 0.268 |
|  | Convulxin | 5,370  (4,740-5,933) | 5,251  (4,769-5,862) | 0.678 |
|  | SFLLRN | 3,253  (2,386-4,230) | 3,279  (2,510-3,928) | 0.949 |
| CD63 | Vehicle | 2,061  (1,984-2,295) | 2,173  (1,925-2,362) | 0.405 |
|  | ADP | 2,726  (2,396-2,918) | 2,786  (2,609-3,134) | 0.201 |
|  | Convulxin | 5,495  (4,517-6,143) | 5,484  (4,839-5,959) | 0.868 |
|  | SFLLRN | 4,387  (3,538-5,218) | 4,574  (3,841-5,083) | 0.708 |
| Bound fibrinogen | Vehicle | 18,766  (14,693-28,274) | 20,225  (13,795-27,774) | 0.865 |
|  | ADP | 44,900  (36,628-62,962) | 48,334  (39,292-62,924) | 0.604 |
|  | Convulxin | 51,756  (44,686-61,003) | 52,766  (40,082-60,526) | 1.00 |
|  | SFLLRN | 38,280  (31,855-56,034) | 43,911  (25,817-57,743) | 0.807 |
