# Supplementary material for: Platelet Dysfunction in Thrombosis Patients Treated with Vitamin K Antagonists and Recurrent Bleeding
Source: PLoS One. 2013 May 28;8(5):e64112. doi: 10.1371/journal.pone.0064112 (PMC3665853; doi:10.1371/journal.pone.0064112)
Supplement: Table S4 — Agonist-induced secretion and integrin activation of platelets from controls and cases. PRP diluted in Hepes buffer was activated with 10 µM ADP, 50 ng/mL convulxin or 15 µM SFLLRN. Flow-cytometric detection of α-granule secretion using FITC-labeled P-selectin, dense-granule secretion with APC-labeled anti-CD63 and platelet fibrinogen binding with FITC-labeled anti-fibrinogen mAb. Data represented as fractions of positive platelets. Medians with interquartile ranges. (DOC) [file pone.0064112.s004.doc]

| **Marker** | **Agonist** | **Controls**  *pos. platelets (%)* | **Cases**  *pos. platelets (%)* | ***P*-value** |
| --- | --- | --- | --- | --- |
| P-selectin | Vehicle | 3.40 (2.88-4.96) | 3.90 (2.61-6.23) | 0.446 |
|  | ADP | 51.4 (43.0-63.6) | 56.2 (48.4-61.6) | 0.366 |
|  | Convulxin | 91.2 (87.0-93.1) | 89.5 (86.2-93.0) | 0.391 |
|  | SFLLRN | 75.0 (65.3-81.2) | 74.3 (68.1-81.5) | 0.848 |
| CD63 | Vehicle | 11.6 (8.99-14.8) | 13.4 (11.1-14.8) | 0.151 |
|  | ADP | 30.4 (22.6-33.6) | 26.6 (25.4-32.7) | 0.732 |
|  | Convulxin | 68.8 (64.1-74.0) | 68.8 (61.2-71.7) | 0.483 |
|  | SFLLRN | 54.4 (42.6-60.0) | 54.8 (44.8-59.7) | 0.807 |
| Bound fibrinogen | Vehicle | 6.80 (4.13-9.69) | 5.34 (4.10-8.73) | 0.282 |
|  | ADP | 71.0 (63.1-82.6) | 74.3 (63.2-80.2) | 0.766 |
|  | Convulxin | 78.9 (75.9-84.0) | 79.8 (76.6-83.7) | 0.698 |
|  | SFLLRN | 71.7 (57.5-79.7) | 62.7 (45.3-79.7) | 0.459 |
